# Editorial: Host-pathogen interactions during pregnancy: mechanisms of maternal and fetal immunity

**DOI:** 10.3389/fimmu.2025.1629528

**Published:** 2025-06-10

**Authors:** Yael Alippe, Vanesa Hauk, Gil Mor, Daiana M. Vota

**Affiliations:** ^1^ Department of Medicine, Infectious Diseases, Washington University in St. Louis, St. Louis, WA, United States; ^2^ Universidad de Buenos Aires, Facultad de Ciencias Exactas y Naturales (UBA-FCEN), Departamento de Química Biológica, Consejo Nacional de Investigaciones Científicas y Técnicas (CONICET)-UBA, Instituto de Química Biológica de la Facultad de Ciencias Exactas y Naturales (IQUIBICEN), Buenos Aires, Argentina; ^3^ C.S. Mott Center for Human Growth and Development, Wayne State University, Detroit, MI, United States

**Keywords:** perinatal inflammation, congenital diseases, maternal infections, immune homeostasis in pregnancy, environmental toxicants

During pregnancy, the maternal immune system must balance tolerance toward the semi-allogeneic fetus with protection against infections. This immune equilibrium is orchestrated by intricate interactions between hormonal and immunological signals at the maternal–fetal interface. Disruptions in this balance can lead to pregnancy complications, including spontaneous abortion, preterm birth, and congenital abnormalities. Despite significant progress, many aspects of immune responses during pregnancy and the mechanisms driving infection-induced adverse outcomes remain unclear. This Research Topic provides novel insights into these complex interactions, offering a deeper understanding of maternal-fetal immunity and highlighting potential therapeutic avenues.

A critical aspect of maintaining immune homeostasis during pregnancy is the modulation of inflammation in response to physiological or pathological challenges. Krupa et al. provide evidence that B lymphocytes play a crucial role in fetal tolerance through secretion of soluble CD83 (sCD83), an anti-inflammatory mediator. Upon bacterial stimulation, decidual and peripheral B cells exhibit distinct capacities for sCD83 secretion, modulated by pregnancy-associated hormones such as estradiol (E2), progesterone (P4), transforming growth factor-beta 1 (TGF-β1), and human chorionic gonadotropin (hCG). While these hormones enhance lipopolysaccharide (LPS)-induced CD83 expression, glucocorticoids inhibit this process, underscoring the complex hormonal regulation that fine-tunes immune responses during pregnancy.

In line with this hormone–immune interplay, Chen et al. explore the role of placental alkaline phosphatase (ALPP), an enzyme highly expressed during pregnancy. Their studies using transgenic mouse models reveal that increased ALPP expression heightens susceptibility to LPS-induced sepsis and disrupts adaptive immune responses. This dual function of ALPP—mediating both tolerance and defense—highlights the intricate balance required for healthy pregnancy outcomes, particularly in bacterial infection contexts.

Expanding upon the concept of immune tolerance, Koenig et al. investigate decidual leukocyte responses to Zika virus (ZIKV) infection in rhesus macaques. Interestingly, the study reports a predominantly anti-inflammatory response characterized by increased CD163 expression on decidual macrophages and minimal pro-inflammatory cytokine production. Contrary to the expected heightened inflammatory response, these findings suggest the decidua actively maintains a tolerogenic environment to minimize fetal harm during viral infections. This research deepens our understanding of decidual immune modulation and identifies potential targets for therapeutic interventions.

The intricate relationship between microbial exposure and pregnancy outcomes is further explored by Einenkel et al., who examine how interactions between *Fusobacterium nucleatum* and maternal immune cells influence inflammation and trophoblast function. While high bacterial loads correlate with severe inflammation and adverse outcomes, lower bacterial burdens induce mild inflammatory responses that may benefit implantation and placentation. This study underscores the importance of identifying microbial thresholds and their immunological impacts, potentially guiding microbiota-targeted interventions to support pregnancy health.

Broader global implications of infection-related adverse pregnancy outcomes are detailed by Qian et al., who provide a thorough pre-pandemic epidemiological analysis of maternal sepsis and maternal infection trends from 1990 to 2019. Their findings reveal a significant overall decrease in global incidence and mortality rates but highlight persistent regional disparities, particularly in the African region. This disparity emphasizes the urgent need for targeted healthcare policies at global, regional, and national levels aimed at equitable access to maternal care and improved management of pregnancy-related infections worldwide.

Focusing on therapeutic interventions, Coward-Smith et al. explore the efficacy of low-dose aspirin (ASA) and its nitric oxide-conjugated derivative (NCX4016) in mitigating vascular inflammation and improving pregnancy outcomes during influenza A virus (IAV) infection. Their findings demonstrate that ASA and NCX4016 effectively prevent IAV-induced vascular dysfunction, reduce systemic viral dissemination, and improve offspring survival rates. These results underscore the therapeutic potential of aspirin beyond its established role in preeclampsia prevention, highlighting its broader clinical relevance in managing infection-associated complications during pregnancy.

Lastly, Stephens et al. offer a comprehensive review on the influence of environmental toxicants, specifically persistent organic pollutants such as 2,3,7,8-tetrachlorodibenzo-p-dioxin (TCDD), on maternal immune function. Activation of the aryl hydrocarbon receptor (AhR) pathway by these toxicants disrupts immune responses essential for maintaining maternal-fetal tolerance and protecting against infections. Epidemiological and experimental studies reviewed by Stephens et al., link such environmental exposures to significant adverse reproductive outcomes, including infertility, placental inflammation, endometriosis, and preterm birth. By emphasizing environmental factors as critical external modulators of immune function, this review complements the findings on immune responses to pathogens during pregnancy explored in this Research Topic, underscoring the need for holistic, integrative approaches to safeguarding maternal and fetal health.

Collectively, the contributions to this Research Topic deepen our understanding of the complex immunological balance essential for sustaining a healthy pregnancy by identifying critical immune pathways and regulatory mechanisms triggered by exposure to pathogens or environmental pollutants, along with potential therapeutic interventions. The insights gained not only expand fundamental knowledge of maternal-fetal immunobiology but also provide pathways toward the development of effective interventions to mitigate infection-related pregnancy complications, ultimately advancing global efforts to improve outcomes for both mothers and their offspring.

